# Surface Acoustic Wave Ammonia Sensors Based on ST-cut Quartz under Periodic Al Structure

**DOI:** 10.3390/s90200980

**Published:** 2009-02-16

**Authors:** Cheng-Liang Hsu, Chi-Yen Shen, Rume-Tze Tsai, Ming-Yau Su

**Affiliations:** Department of Electrical Engineering, I- Shou University, Kaohsiung County, 84001, Taiwan

**Keywords:** Rayleigh SAW, space harmonic method, ammonia, polyaniline/WO_3_

## Abstract

Surface acoustic wave (SAW) devices are key components for sensing applications. SAW propagation under a periodic grating was investigated in this work. The theoretical method used here is the space harmonic method. We also applied the results of SAW propagation studied in this work to design a two-port resonator with an Al grating on ST-cut quartz. The measured frequency responses of the resonator were similar to the simulation ones. Then, the chemical interface of polyaniline/WO_3_ composites was coated on the SAW sensor for ammonia detection. The SAW sensor responded to ammonia gas and could be regenerated using dry nitrogen.

## 1. Introduction

The release of various pollutants into the atmosphere has caused global environmental issues. Most of the ammonia in the atmosphere is emitted by human activity directly or indirectly. There are three major sources that emit ammonia in the atmosphere. The direct depositions of ammonium and nitrate salts in the form of dissolved dust or particles in rain water are the first source. The second source is ammonification that is performed by the combination of bacteria and fungi within the nitrogen cycle. The third source is combustion from chemical industry. Exposure to high ammonia concentration can result in life-threatening situation. Moreover, the ammonia aerosols have a sun-blocking function, so these clouds of smog produce a temperature reducing effect. Therefore, the detection of ammonia gas is an important task. There are many kinds of gas sensors to have been widely used for ammonia detection in the environment, including acoustic wave-based sensors, metal-oxide sensors, catalytic detectors, polymer sensors, and optical sensors. Metal-oxide sensors and catalytic detectors must be operated at high temperatures, up to 250-600°C. Polymer sensors in general suffer from a lack of reversibility. Optical sensors are expensive and are not easily miniaturized. With the advanced microfabrication and signal processing techniques, surface acoustic wave (SAW) devices offer significant advantages of real-time, sensitive, and stable responses [1,2]. Therefore, their applications in the development of gas sensors have received considerable attention. The common wave modes applied in SAW gas sensors are usually Rayleigh SAW and shear horizontal SAW. In this study, we report a SAW gas sensor for monitoring ammonia near intensive farming locations. Concentration levels of ammonia near intensive farming sites can be higher than the allowable exposure limits. This may lead to unhealthy situations for both farmers and animals, so its early detection is highly desirable. Sensors for this application must be able to detect ppm-level ammonia at room temperature [3]. In all piezoelectric substrates, ST-cut quartz with high temperature stability at room temperature has been widely utilized to design SAW devices [4,5]. Therefore, we choose ST-cut quartz, whose wave mode is Rayleigh SAW, to be the substrate, and we develop a SAW sensor based on ST-cut quartz to detect ammonia in a range of 26-85 ppm at room temperature.

The basic structures of the SAW devices consist of a piezoelectric substrate and periodic metal gratings. It is important to investigate the propagation of the SAW under a periodic metal grating in order to efficiently design an SAW device. Due to the complexity of the boundary condition, it is not easy to study the propagation of the SAW with a consideration of the mass loading effect of periodic metal grating [6-8]. Several analysis techniques have been developed for this purpose, including the finite element method (FEM) [9-11], the Green function method [12-16], and the space harmonic method (SHM) [17,18].

FEM is well suited for analysis of finite dimension problems with arbitrary geometries. The perturbation of SAW propagation requires meshing the electrode and the substrate, which results in extensive computer time demands, so FEM must reduce the models, complexity or size by neglecting special properties and FEM is calculated in the limit of infinitely thin electrodes, where the mass loading effect is not taken into account. In order to overcome this disadvantage, advance development of the FEM, i.e. the finite-element/ periodic boundary conditions (FE/PBCs) and the finite element method/boundary element method (FEM/BEM), for the analysis of SAW propagation have been presented. The use of PBCs in the FEM allows the reduction of the periodic simulation domain to one base cell, but it deals with the calculation of the phase constant only. Hofer *et al.* [9] proposed two different methods, which are the Schur-Complement method and a method with inner nodes, to analyze wave propagation in periodic piezoelectric SAW structures and have delivered different numerical properties. They have also shown how to compute the charge distribution of periodic SAW structures with the aid of the new PBCs. The FEM/BEM computation model can solve a large linear system for each frequency point. Laude *et al.* [10] proposed an asymptotic waveform evaluation technique to obtain an approximate solution of the linear system that is valid over a large frequency bandwidth and vastly reduces the computation time. Gamble *et al.* [11] take into account the effect of finite electrode resistance, which they used FEM to model the electrodes, BEM to the substrate, and pulse functions to approximate surface charge density, surface free charge, and surface potential. They have made a comparison between theory and experiment for the aluminum electrode on LiTaO_3_ with good consistency.

Early, the concept of Green function method has been widely used to solve the electromagnetic problems [19-21]. This approach was ever limited by its complexity and computational cost. Now by improving the analysis and combining it with more powerful computers, this makes the Green function method usable to analyze SAW propagations. Peach and Xu [14] have used non-linear programming techniques directly to a Green function mode, and have applied this successfully to analyze both SAW and leaky SAW. Laude *et al.* [15] discussed the computation of the 2-D harmonic spatial-domain Green function at the surface of a piezoelectric half-space. Ventura *et al.* [16] combined FEM with the Green function method on analysis of periodic SAW structure to the calculation of reflection and scattering parameters.

SHM can integrate along boundaries so it is easy to apply to arbitrarily periodic shaped structures. Therefore, we used SHM to investigate the Rayleigh SAW propagating under the periodic metal grating in this work. The boundary integral equations were derived from the method of weighted residuals for a period of each region, such as substrate, metal, and free space, and were solved to satisfy the periodic boundary conditions. The metal grating in this work was Al. Aluminum is a common material for gratings because of its well developed manufacture techniques.

By using the coupling-of-modes (COM) theory [22-24], we design a two-port resonator with an Al grating on ST-cut quartz to prove the accuracy of the propagation characteristics studied in this work. COM theory is a powerful analysis method in designing SAW devices. First it is essential to determine the COM parameters, which are the self-coupling coefficient κ_11_, the reflection coefficient κ_12_, and the transduction coefficient ζ. The COM parameters can be obtained from the dispersion curves and stopband properties of SAW propagating. This two-port resonator was then used in this work as an ammonia sensor.

The chemical interface was a polyaniline/WO_3_ nanocomposite. Polyaniline film is easily prepared from aqueous solutions and is an effective interface for detecting ammonia [25-34]. The WO_3_ material has also shown good responses to ammonia [35,36]. Sensors based on polyaniline/WO_3_ nanocomposite have been reported to detect humidity and hydrogen [37,38]. The combination of polyaniline and WO_3_ has the potential to improve sensitivity of detection at room temperature and retain the advantages of its constituents with increased surface functionalities for gas detection. In addition, selectivity and long term stability of the sensors may be optimized by controlling the volume ratio of polyaniline to metal oxide nanoparticles. The gas sensor developed in this work has two advantages: the polyaniline/WO_3_ nanocomposite layer can sensitively interact with ammonia, and the SAW device is fast output, high sensitivity, good reliability, and low cost.

The remainder of this paper is divided into three sections. In the section that follows the concept of SHM is introduced. The Rayleigh SAW propagation under periodic gratings on Al/ST-cut quartz structure is analyzed by SHM. Then, it calculates the dispersion curves and the COM parameters. In Section 3, the SAW sensors, based on Al/ST-cut quartz with polyaniline/WO_3_, are realized and used to detect to ammonia at levels of 26.15-87.80 ppm. Finally, a brief summary is given in Section 4.

## 2. Theoretical Approach

**Figure 1.**
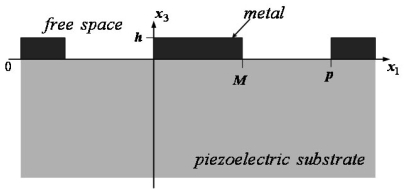
The coordinate system.

The SHM method can analyze the propagation under periodic gratings. It only requires the analysis of one period and expresses the displacements and the electric potential as the sum of space harmonic. Figure 1 shows the coordinate system in this work. The SAW propagates in the *x*_1_ direction with period *p*. Herein, *M* and *h* are width and thickness of the metal grating, respectively.

In each region, the displacement *U_i_* and the electric potential *φ* are written as: substrate:

(1) $$U_{i}^{s\prime }=\sum_{m=-\infty }^{\infty }\sum_{n=1}^{4}A^{\left(m,n\right)}\beta_{i}^{\left(m,n\right)}\exp\left\{j\frac{\pi }{p}\left[\gamma \alpha^{\left(m,n\right)}x_{3}+\left(\gamma +2m\right)x_{1}-f\left(2p\right)t\right]\right\},(i=1,2,3)$$

(2) $$U_{4}^{s\prime }=\phi^{s\prime }=\sum_{m=-\infty }^{\infty }\sum_{n=1}^{4}A^{\left(m,n\right)}\beta_{4}^{\left(m,n\right)}\exp\left\{j\frac{\pi }{p}\left[\gamma \alpha^{\left(m,n\right)}x_{3}+\left(\gamma +2m\right)x_{1}-f\left(2p\right)t\right]\right\},$$

metal:

(3) $$U_{i}^{m\prime }=\sum_{m=-\infty }^{\infty }\sum_{n=5}^{10}A^{\left(m,n\right)}\beta_{i}^{\left(m,n\right)}\exp\left\{j\frac{\pi }{p}\left[\gamma \alpha^{\left(m,n\right)}x_{3}+\left(\gamma +2m\right)x_{1}-f\left(2p\right)t\right]\right\},\left(i=1,2,3\right)$$

free space:

(4) $$U_{4}^{f\prime }=\phi^{f\prime }=\sum_{m=-\infty }^{\infty }A^{\left(m,0\right)}\beta_{4}^{\left(m,0\right)}e\left\{j\frac{\pi }{p}\left[\gamma \alpha^{\left(m,0\right)}x_{3}+\left(\gamma +2m\right)x_{1}-f\left(2p\right)t\right]\right\},$$

where *m* denotes the space harmonic and *n* is the index of the partial waves inside the layer. *A*^(^*^m,n^*^)^ is the weighting factor, *α*^(^*^m,n^*^)^ is the decay factor in the *x_3_* direction, *β_i_*^(^*^m,n^*^)^ is the component of the normalized eigenvector corresponding to the *m-th* space harmonic, *γ* is the normalized wave number, *f* is the frequency, and *t* is the time.

The wave equations under conditions of a quasi static approximation as follows: substrate:

(5) $$T_{ij,j}^{s\prime }=c_{\mathrm{ijkl}}^{s\prime }U_{k,lj}^{s\prime }+e_{\mathrm{kij}}^{s\prime }U_{4,kj}^{s\prime }=\rho^{s\prime }{\ddot{U}}_{i}^{s\prime },\left(i,j,k.l=1,2,3\right)$$

(6) $$D_{i,i}^{s\prime }=e_{\mathrm{ikl}}^{s\prime }U_{k,lj}^{s\prime }-\varepsilon_{ik}^{s\prime }\phi_{,ik}^{s\prime }=0$$

metal:

(7) $$T_{ij,j}^{m\prime }=c_{\mathrm{ijrs}}^{m\prime }U_{r,sj}^{m\prime }=\rho^{m\prime }{\ddot{U}}_{i}^{m\prime }$$

free space:

(8) $$D_{i,i}^{f\prime }=-\varepsilon_{0}\phi_{,ii}^{f\prime }=0$$

where *T_ij_* is the stress, *D_i_* is the electric displacement, *c* is the elastic tensor, *e* is piezoelectric tensor, *ε* is the dielectric tensor, and *ρ* is the mass density. In Equations (5)-(8), a dot denotes differentiation with respect to time and a comma denotes differentiation with respect to a space coordinate.

Substituting Equations (1)-(4) into the wave equations, the *α*^(^*^m,n^*^)^ and *β_i_*^(^*^m,n^*^)^ can be obtained. In general, four *α*^(^*^m,n^*^)^ are selected according to physical considerations in the substrate region, six *α*^(^*^m,n^*^)^ are applied in the metal region, and one *α*^(^*^m,n^*^)^ is selected according to physical considerations in the free space.

**Figure 2.**
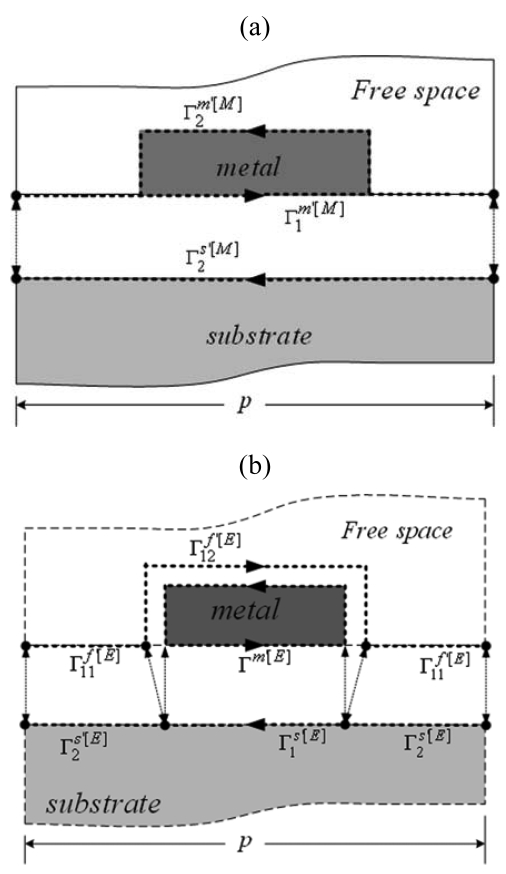
Boundary conditions for theoretical analysis: (a) Mechanical boundary conditions. (b) Electrical boundary conditions.

The weighting factors *A*^(^*^m,n^*^)^ are determined from the mechanical and electrical boundary conditions shown in Figure 2. For the mechanical boundary conditions, the displacements and the stress across the boundary between the substrate and the metal region must be continuous, and the stress across the boundary between the metal region and the free space must be zero. For the electrical boundary conditions, the potential and the normal component of electric displacement must be continuous across the boundaries, and the potential must be a constant value on the metal region.

In each region, the boundary conditions can be expressed by boundary integral equations in the following: substrate:

(9) $$\begin{array}{l} \int_{\Gamma_{2}^{s\prime \left[M\right]}}\text{Re}\left[\left(P_{i}^{m\prime *}-P_{i}^{s\prime *}\right)\delta U_{i}^{s\prime }\right]d\Gamma +\int_{\Gamma_{2}^{s\prime \left[E\right]}}Re\left[\left(P_{4}^{f\prime *}-P_{4}^{s\prime *}\right)\delta \phi^{s\prime }\right]d\Gamma \\ +\int_{\Gamma_{1}^{s\prime \left[E\right]}}Re\left[\left(\phi^{s\prime }-V_{0}\right)\delta P_{4}^{s\prime *}\right]d\Gamma =0 \end{array}$$

metal:

(10) $$\int_{\Gamma_{1}^{m\prime \left[M\right]}}Re\left[\left(U_{i}^{m\prime }-U_{i}^{s\prime }\right)\delta P_{i}^{m\prime *}\right]d\Gamma +\int_{\Gamma_{2}^{m\prime \left[M\right]}}Re\left[-P_{i}^{m\prime *}\delta U_{i}^{m\prime }\right]d\Gamma =0$$

free space:

(11) $$\int_{\Gamma_{11}^{f\prime \left[E\right]}}\text{Re}\left[\left(\varphi^{f\prime }-\varphi^{s\prime }\right)\delta P_{4}^{f\prime *}\right]d\Gamma +\int_{\Gamma_{12}^{f\prime \left[E\right]}}Re\left[\left(\varphi^{f\prime }-V_{0}\right)\delta P_{4}^{f\prime *}\right]d\Gamma =0$$

For the electrical boundary conditions, there are two kinds of electrical connections that are shorted and open gratings. In the shorted case, the individual metal strips are grounded. Thus the electric potential on the metal strips is zero. In the open case, each metal strip is electrically isolated. Therefore, the total charge on the metal region *Q^m′^* must be zero.

Then, we get a matrix equation in the real and imaginary parts of the voltage *V_0_* and the weighting factors *A*^(^*^m,n^*^)^, the matrix equation is as follows:

(12) C⋅Y=0

where

(13) Y=[Re(V0),Im(V0),Re(A(−∞,0)),Im(A(−∞,0)),…,Re(A(m,n)),Im(A(m,n)),‥‥,………………‥,Re(A(∞,10)),Im(A(∞,10))]T

The exact solution can be obtained by evaluating the normalized wave number *γ* and the normalized frequency *f*·(*2p*), which satisfy the condition *det*|C| = 0. The stopband frequencies converge in a similar manner as described in [18] when m increases. Hence, the number of space harmonic m was truncated to 4 in this work.

**Figure 3.**
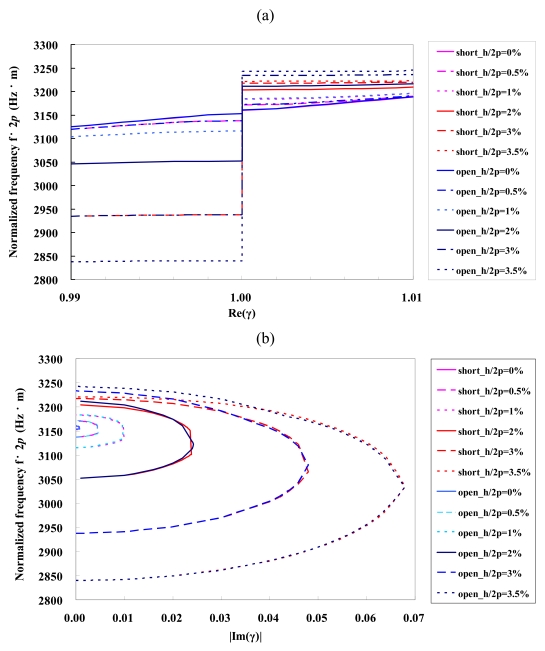
Dispersion curves of Rayleigh wave under the shorted and open grating on *Al*/ST-cut quartz at *M/p* = 0.5: (a) The real part of normalized wave number. (b) The imaginary part of normalized wave number.

The dispersion curves of the Rayleigh wave under shorted and open Al grating on ST-cut quartz as a function of the normalized *Al* thickness, *h/2p*, are illustrated in Figure 3, respectively. The stopband width increases with increasing Al strip thickness. The stopband width for the shorted grating at *h*/2*p* = 3.5% is about 57.8 times as wide as that at *h*/2*p* = 0. The stopband width for the open grating is wider than that for the shorted grating. It indicates enhanced reflectivity for the open grating. The variation of the upper stopband edge with Al strip thickness is less than that of the lower stopband edge. This observed phenomenon has a relatively complex nature, due to a variety of factors acting simultaneously [39]. Increasing Al strip thickness, it results in the increasing mass loading effect and causes a decrease in the velocity. The lower velocity of propagation in the aluminum strips also causes the slowing down effect. Moreover, the reflection induced contributes to a further slow wave at the lower stopband edge and to an increase in the propagation velocity at the upper stopband edge. Therefore, these reasons lead to the sensitivity of the lower stopband edge is greater than that of the upper stopband edge. The imaginary part of the normalized wave number also increases with the Al strip thickness. It indicates the thick Al strip thickness results in a larger mass loading effect and induces a large attenuation.

The COM theory is based on the concept that the progressing wave and counter-progressing wave couple with each other in periodic structures. The parameters used in the COM theory include the mutual coupling coefficient *κ_12_*, the self-coupling coefficient *κ_11_*, and the transduction coefficient *ζ*. These parameters can be determined from the four stopband edge frequencies (*f_us_, f_ls_, f_uo_*, and *f_lo_*) of the dispersion curves for shorted and open gratings [40]. They are summarized as,

(14) $$\kappa_{11}\cdot 2p=2m\pi \left(1-\frac{f_{ls}+f_{us}}{2mf_{o}}\right)$$

(15) $$\left|\kappa_{12}\cdot 2p\right|=\pi \frac{f_{us}-f_{ls}}{f_{0}}$$

(16) $$\frac{{\left|\zeta \left(2p\right)\right|}^{2}}{\omega_{0}C_{s}}=\frac{\pi }{2}\frac{1}{f_{0}^{2}}\left\{\left(f_{u0}^{2}+f_{l0}^{2}+f_{u0}f_{l0}+f_{us}f_{ls}\right)-\left(f_{us}+f_{ls}\right)\left(f_{u0}+f_{l0}\right)\right\}$$

where *f*_0_ = *v_f_*/(2*p*) is the Bragg frequency (*v_f_* the phase velocity for the free surface) and *C_s_* is the static capacitance per grating pair.

**Figure 4.**
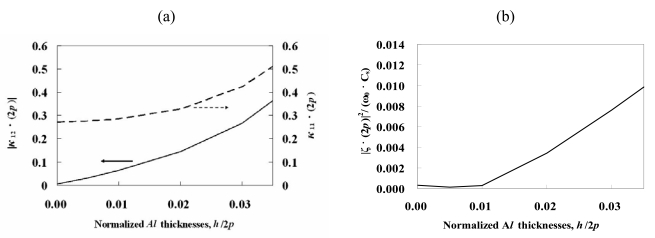
The COM parameters under the Al periodic grating on ST-cut quartz: (a) |*κ_12_*·2*p*| and *κ_11_*·2*p* and (b) |*ξ*(*2p*)|*^2^*/(*ω_0_C_s_*).

Figure 4 shows the relationships of the parameters |*κ*_12_ · 2*p*|, *κ*_11_ · 2*p* and |*ξ*(2*p*)|^2^/(*ω*_0_*C_s_*), which were derived from Equations (14)-(16), and Al thickness. Figure 4(a) shows that |*κ*_12_ · 2*p*| increases as the metal grating thickness *h*/2*p* increases. This indicates that an increasing thickness of the Al grating increases the reflectivity. Hence, the thicker Al gratings with large effective reflection are suitable for the application of the resonator with a small size. *κ*_11_ · 2*p*, associated with the phase shift per period, increases with increasing the metal grating thickness *h*/2*p*. Figure 4(b) shows that the |*ξ*(2*p*)|^2^/(*ω*_0_*C_s_*) parameter is sensitive to Al thickness and is a linear function of the Al grating's thickness above *h*/2*p* = 1%.

## 3. Experimental Procedures and Results

**Figure 5.**
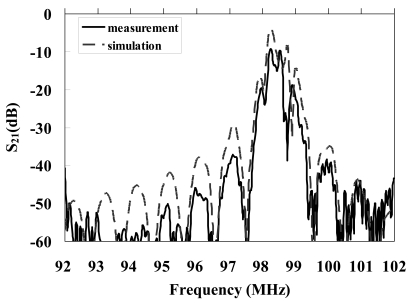
Frequency responses of two-port SAW resonator.

We designed and realized a two-port resonator with a shorted grating to investigate experimentally the accuracy of the theoretical results. Each of the Al input and output interdigital transducers (IDT) has 100 finger pairs, the period *p* is 16 μm, each of reflector has 150 Al strip gratings, aperture of IDT is 960 μm, the thickness of Al is 320 nm, the metallization ratio *M/p* is 0.5, and the center-to-center distance between the IDTs is 600 μm. Polyimide covered the surface of the IDTs and the reflector to form a protective layer. The operating frequency of the resonator was 98.32 MHz. Figure 5 illustrates the frequency response of the two-port resonator. It shows that measurement data are similar to the simulation one.

**Figure 6.**
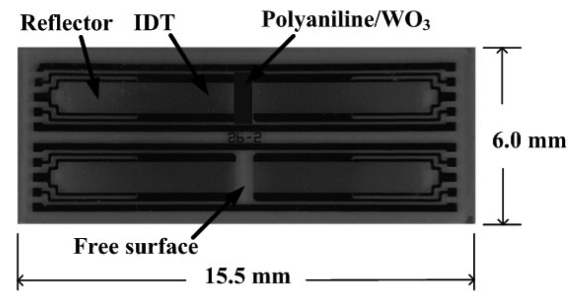
The photograph of a dual-device configuration.

The chemical interface was polyaniline/WO_3_ composite coated on the space between the input and output IDTs of the two-port resonator. This study made use of a dual-device configuration shown in Figure 6 to reduce interference from the environment. A non-coated resonator was used as a reference, and a coated resonator was used as a sensing device. The SAW resonator was connected to an RF electronic oscillator circuit to generate RF signals. The responses to ammonia were measured by the frequency shift Δ*f* that is the frequency difference between the coated resonator and the non-coated resonator.

**Figure 7.**
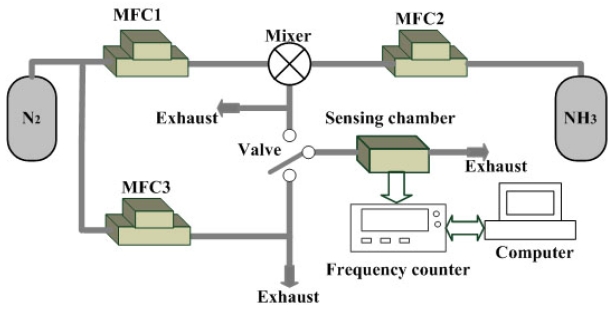
Schematic diagram of a gas testing system for ammonia detection.

Dry nitrogen was the carrier gas. Mass flow controllers (Sierra, USA) were used to control and monitor the flow. The gaseous ambience was maintained at a flow rate of 110 mL/min. To sense for ammonia, the dry nitrogen and ammonia were mixed in various ratios. Then the dual-device configuration was put into a sealed 5 cm^3^ sensing chamber. All detections were performed at room temperature. A frequency counter connected to a computer system via a RS-232 interface board monitored the frequency differences between the sensing device and the reference device. The sensing system is illustrated in Figure 7.

**Figure 8.**
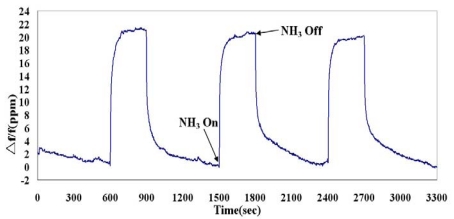
Frequency responses of the SAW sensor to 77 ppm ammonia.

The real-time responses of the SAW sensor to 77 ppm ammonia in dry nitrogen are shown in Figure 8. The sensor detected the presence of ammonia gas after ammonia turned on. The response of the sensor reversibly returned to its original condition when ammonia turned off and dry nitrogen purged. Moreover, each gas on/off cycle presents similar response, revealing repeatable detection property. Detecting to 77 ppm ammonia, the frequency shift is 20.1 ppm, the noise level is 0.18 ppm, and a signal-to-noise ratio is 111.7.

**Figure 9.**
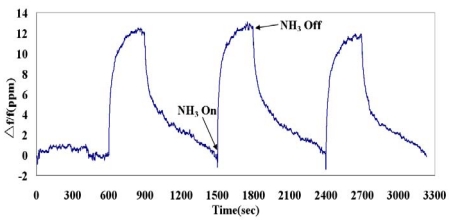
Frequency responses of the SAW sensor to 40 ppm ammonia.

Figure 9 illustrates the frequency responses of the SAW sensor to 40 ppm ammonia in dry nitrogen. This sensor was sensitive to 40 ppm ammonia and also presented the reversible and repeatable responses. The frequency shift is 11.6 ppm, the noise level is 0.16 ppm, and a signal-to-noise ratio is 72.5.

As the gas absorption, the frequency shift Δ*f/f* of the SAW sensor can be described by the following [41]:

(17) $$\frac{\Delta f}{f_{0}}≅\frac{\Delta v}{v_{0}}=-\omega h\left[c_{1}\left(\rho -\frac{\mu }{v_{0}^{2}}\right)+c_{2}\rho +c_{3}\rho \right]+\omega h\left[\frac{4\mu }{v_{0}^{2}}\frac{\lambda +\mu }{\lambda +2\mu }\right]-\left[\frac{K^{2}}{2}\frac{1}{{\left(v_{0}C_{s}/\sigma_{s}\right)}^{2}+1}\right]$$

where *c_i_* are SAW-chemical interface coupling parameter.*ρ, h, σ_s_, μ, λ* are density, thickness, sheet conductivity, shear modulus, and Lame constant of the chemical interface, respectively. The three terms on the right of Equations (17) indicate the contributions of change in mass loading, elastic effect and acoustoelectric effect, respectively, to total changes in frequency shift of the SAW sensor.

**Figure 10.**
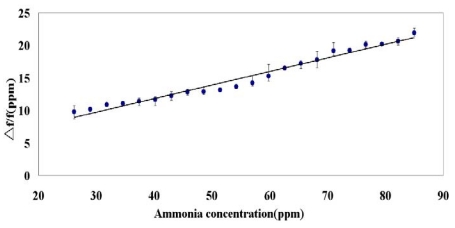
Responses of the SAW gas sensor to the concentration of ammonia.

Figure 10 shows an ammonia concentration dependence of the frequency shift of the SAW sensor. Systemically increasing ammonia concentration caused the positive frequency shift to increase linearly. This phenomenon can be explained by considering the perturbation mechanism. According to the perturbation mechanism [41], the magnitude of the negative frequency shift increases as the concentration of analyte increases, while the change in the elastic modulus effect is negligible compared to the change in the mass loading effect and acoustoelectric effect. However, the magnitude of the negative frequency shift decreases as the concentration of analyte increases when the change in the elastic modulus effect is not neglected or is comparable to the change in the mass loading effect and acoustoelectric effect. A positive frequency response occurs as the elastic modulus effect significantly increases and exceeds the change in mass. In Figure 10, the positive frequency responses show that the elastic effect exceeds the change in mass loading and acoustoelectric effect. The sensitivity is defined as (change in sensor output)/(change in gas concentration). In this work, the SAW sensor produced the frequency changes of 1,160.8 Hz to respond the concentration of 26–85 ppm ammonia. In reference [29], the film that is polyaniline synthesized by poly(4-styrenesulfonate-co-maleic acid) had the resistance changes of 3,612.5 kΩ to respond the concentration of 5–250 ppm ammonia. In reference [30], an optical sensor based on the polyaniline film showed the voltage changes of 0.013 V to respond the concentration of 200-1,000 ppm ammonia. Therefore, the SAW sensor reported in this work has the sensitive responses to 26–85 ppm ammonia.

## 4. Conclusions

In this work, SHM was applied to analyze the Rayleigh SAW propagating under the periodic *Al* grating on the ST-cut quartz. The stopband width of the dispersion curves of the Rayleigh SAW increases with increasing Al strip thickness. The open grating shows the enhanced reflectivity. |*κ*_12_ · 2*p*|, *κ*_11_ · 2*p* and |*ξ*(2*p*)|^2^/(*ω*_0_*C_s_*) parameter are sensitive to Al thickness. The SAW sensor, which was designed by using the propagating analysis in this work, coated with polyaniline/WO_3_ composites exhibited the response to ammonia at room temperature. The response could be recovered by dry nitrogen easily and presented the repeatability. Detecting to 77 ppm ammonia, the frequency shift was 20.1 ppm, the noise level was 0.18 ppm, and a signal-to-noise ratio was 111.7. The positive frequency shift increased linearly with increasing ammonia concentration. It shows that the elastic effect exceeds the change in the mass loading and acoustoelectric effects. In future, we will keep studying the selectivity and long-term stability of this sensor for practical application.
